# PSLite Online: A Portable, Modern Web Application to Analyze Top‐Down Mass Spectrometry Fragmentation Data

**DOI:** 10.1002/jms.70037

**Published:** 2026-02-12

**Authors:** Ryan T. Fellers, Joseph B. Greer, Bryan P. Early, Michael A. R. Hollas, Matthew T. Robey, Neil L. Kelleher, Kenneth R. Durbin

**Affiliations:** ^1^ Proteinaceous Inc. Evanston Illinois USA; ^2^ Department of Chemistry Northwestern University Evanston Illinois USA

**Keywords:** bioinformatics, top‐down mass spectrometry, top‐down proteomics

## Abstract

*PSLite Online* is a modern, web‐based application designed to facilitate the analysis of top‐down mass spectrometry fragmentation data, particularly for targeted proteoform studies. As a successor to the Windows‐only ProSight Lite, PSLite Online retains and expands upon its core functionalities while offering platform independence and enhanced usability through a responsive, browser‐based interface. Users can load or manually define proteoforms, apply a wide range of modifications, and visualize fragmentation coverage in real time. Key new features include ProForma integration, correction for common mass deconvolution errors, SVG export, and cloud‐based session sharing via persistent URLs. The application leverages modern web technologies and component libraries to deliver a lightweight, installable progressive web app (PWA) experience. Here, we show that PSLite Online can be used for the characterization of antibody subunits with glycan modifications. This solution broadens accessibility for proteomics researchers by eliminating installation barriers and enabling seamless collaboration across devices and platforms. PSLite Online is freely available at https://pslite.proteinaceous.net.

## 1 Introduction

The study of proteins using tandem mass spectrometry involves the matching of fragment masses to a set of candidate sequences, with peptides for bottom‐up proteomics and proteoforms for top‐down proteomics [1]. For large‐scale experiments focused on discovery, a sizeable search space is generated and interrogated systematically [2, 3]. However, many experiments are much more narrow in focus, targeting only a single proteoform or perhaps a single proteoform family [4] with a diverse set of modifications. To support these targeted studies, we have developed PSLite Online, a free, web‐based extension of the popular ProSight Lite [5] Windows application. A web‐based version of ProSight Lite provides numerous advantages, including simplifying the installation process and broadening its reach to any device with a standard web browser.

## 2 Results and Discussion

**FIGURE 1:**
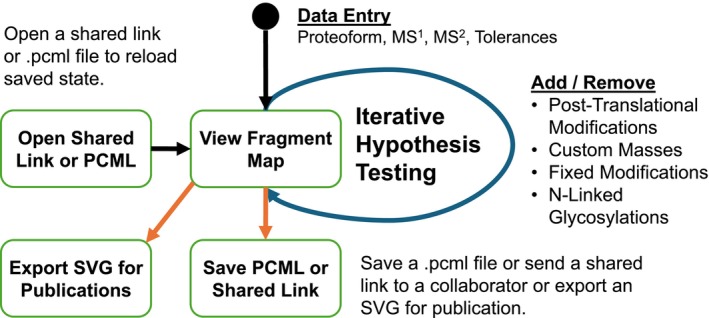
Overview of the PSLite Online workflow. Users can optionally load existing data from a ProSight Lite .pcml file or shared link, modify the proteoform composition or experimental conditions to view changes in matching fragments and scores, and export results to .pcml files, shared links, or fragment map SVGs.

**FIGURE 2:**
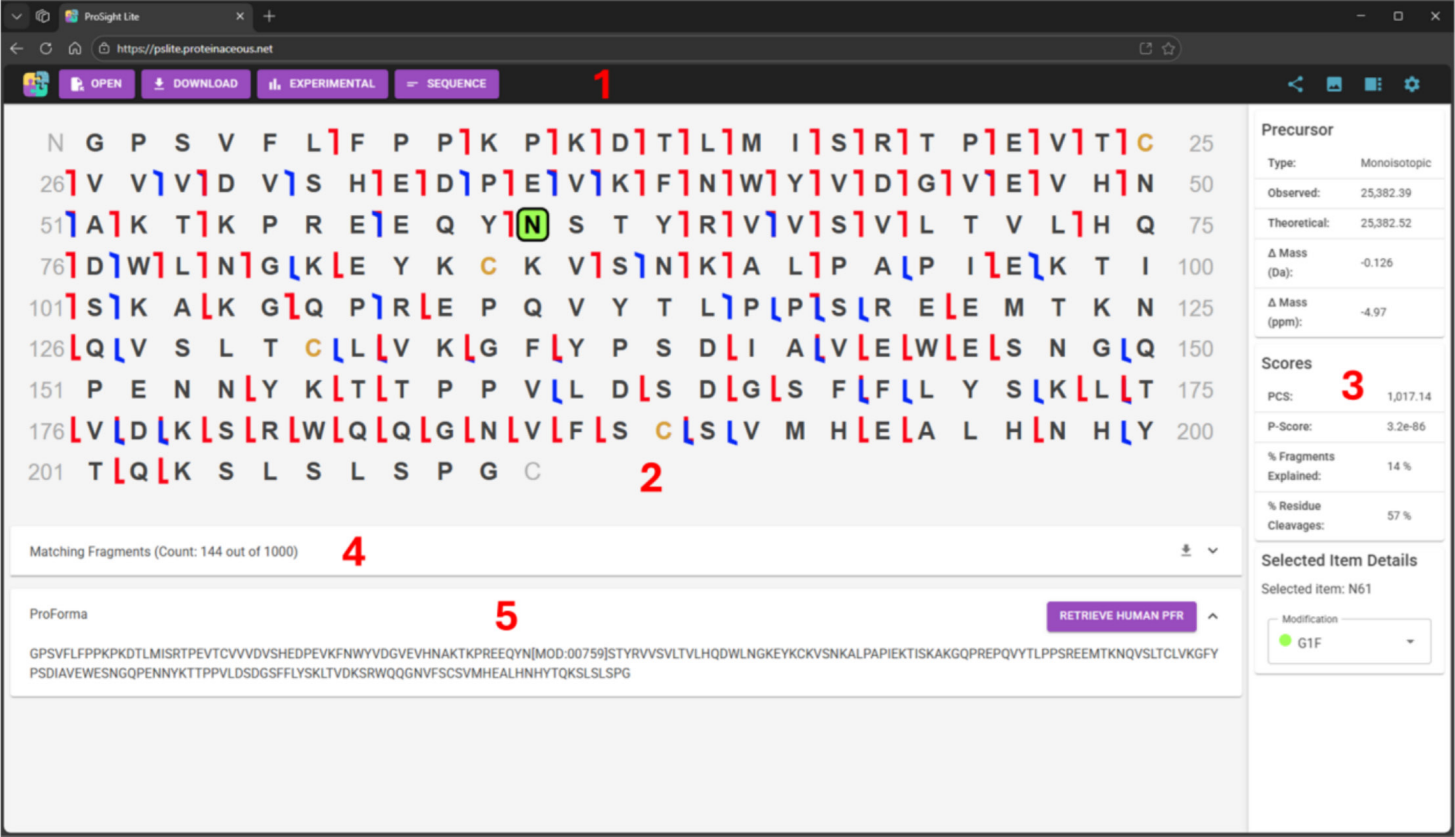
Fragment map of a glycosylated antibody subunit. The new web‐based interface includes (1) a menu bar, (2) a fragment map showing matching fragment ions, (3) mass differences, sequence coverage, and scores, (4) matching fragments details in a grid, and (5) a ProForma sequence for the proteoform.

The basic workflow and features (Figure 1) of the original Windows‐only version have been reimplemented and expanded upon. To begin, the user must populate the state of the application by opening an existing ProSight Lite proteoform characterization markup language (.pcml) file (XML format for legacy version), launching from a shared link (see below for more detail), or entering mass spectral observations and a theoretical proteoform manually. At this point, the user may test different hypothetical proteoforms by adding or removing modifications and observing the changes in the various scoring metrics (Figure 2, #3). An extensive list of possible modifications (post‐translational modifications, fixed modifications, N‐linked glycosylations, and custom mass modifications) is available in this new web‐based version. Once the user is satisfied with their proteoform and fragmentation coverage, they may save the current state in .pcml format, export a publication‐quality SVG (scalable vector graphic) image of the fragment map, or create a shareable link that can be sent to a colleague.

Figure 2 displays the main user interface using the new light mode theme. The interface is similar to the original ProSight Lite interface. When a change is made to the candidate proteoform (by selecting an amino acid and changing the modification on the bottom of the right panel), the fragment map, matching fragments grid, masses, scores, and ProForma [6] are all immediately updated (Figure 2, #2–5). The menu bar (Figure 2, #1) now features new options on the right‐hand side: create a sharable link, export SVG fragment maps, toggle the right panel, and settings. The settings popup allows the user to switch between light and dark mode and set the number of amino acids per line (20, 25, 30, 40, 50, or automatic), which is important when trying to get an exported SVG to fit into a preferred aspect ratio. A section dedicated to ProForma has been added under the matching fragment grid to both show the current proteoform and quickly allow a user to generate a proteoform record (PFR) entry for that proteoform with a set of common species (human, mouse, rat, *E. coli* ) using the Proteoform Registry [7]. Users can also input their proteoform information using a limited set of ProForma features via the Sequence Dialog. The application currently converts formulas to mass shifts but does not support unlocalized, labile, and range modifications. To handle off‐by‐*n* isotoping errors from deconvolution algorithms, one can elect to consider a number of isotope mass shifts to match against. These additional shifts are factored into the P‐score and PCS as additional ion types to keep the scores from being artificially improved. Each off‐by‐one mass shift will add 2 ion types for every selected ion type. Finally, the application can now function in a “theoretical mode” where only the theoretical proteoform is supplied and the intact mass is displayed. A summary of features differences between the original version and the new version is shown in Table 1.

**TABLE 1:** A comparison of features from both versions of PSLite.

Feature description	Original	PSLite Online
Matching fragment map and detailed data grid	✔	✔
Adjustable modifications for each amino acid	✔	✔
Experimental and sequence inputs	✔	✔
Support for .pcml file format	✔	✔
Mass differences and scores	✔	✔
Light and dark theme		✔
Web based/multiple OS support		✔
Handle off‐by‐*n* isotoping errors		✔
Shareable links		✔
Adjustable number of amino acids per line		✔
Basic ProForma support		✔
Proteoform record (PFR) generation		✔

**FIGURE 3:**
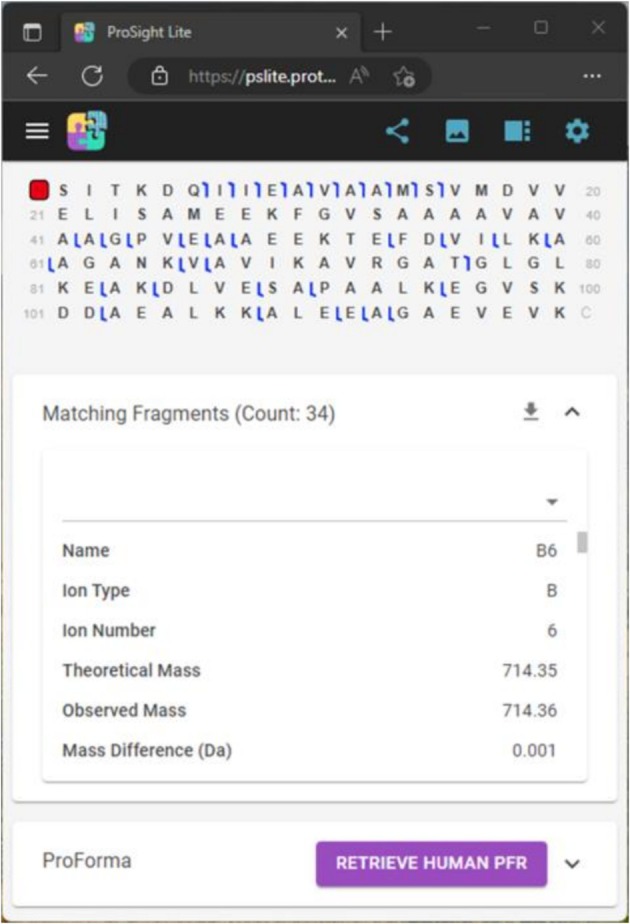
The responsive user interface scales to the device dimensions, allowing use on tablets and mobile devices.

Moving to a web‐based solution enables the application to support a much wider variety of devices (e.g., Mac, iPhone, Android, Linux) and to create the responsive and connected features that most expect from a modern, lightweight application. Our user interface framework allows us to easily support responsive web design (i.e., the application adjusts to fit the device) as well as dark and light mode theming. As shown in Figure 3, when the application is forced into a small size, the top menu collapses to a flyout menu (also known as a hamburger menu), the right panel collapses to an optional flyout, the fragment map automatically shows fewer amino acids per line, and the matching fragment grid turns into a property display control. We also added a welcome dialog with an embedded introduction video, created shareable links by saving application state in the cloud (stored in Azure Blob storage for 1 year), and updated installation of the application to use the progressive web application (PWA) standard. When the PWA version is installed, interactions with the operating system for the original version (e.g., file associations, pinning to taskbars, etc.) are supported.

Overall, PSLite Online provides easy access to top‐down mass spectrometry data analysis through a simple web browser interface. As the value of proteoform characterization continues to be realized, software that improves accessibility and lowers the barrier to entry for proteoform analysis is important. PSLite Online provides a straightforward interface for viewing results that can be shared and used by a wider audience than only mass spectrometry experts. Through its ProForma sequence integration, the language of the proteoform is front and center, bringing more precision to result descriptions. With its host of features, PSLite Online will help drive the next wave of top‐down proteoform innovation and discovery.

## 3 Methods

PSLite Online was implemented in C# 13 and .NET using a Blazor WebAssembly single page application (SPA) and Visual Studio 2026. Most of the functionality (algorithms, user interfaces, etc.) is provided by the MudBlazor Component Library, custom code (.NET 8 or .NET Standard 2.1), and a number of other .NET projects (TopDownProteomics, MathNet. Numerics, DocumentFormat.OpenXML, W8lessLabs.Blazor.LocalFiles, Blazored.LocalStorage, and Quadruple). The fragment map for Figure 2 was generated using data from Oates et al. [8] (MassIVE data repository number MSV000095301) and shows the Fc/2 portion of NIST mAb fragmented using EThcD and PTCR with a G1F glycan at N61. The neutral masses were generated using the THRASH algorithm in the TDValidator module of ProSight Native [9] v1.0.25265.1 with a signal‐to‐noise cutoff of 10 and a minimum RL value of 0.95. Only the 1000 most intense fragments were included.

PSLite can be freely used and accessed at https://pslite.proteinaceous.net under the terms described in the settings menu with no claim to the works or results produced. Questions can be sent to info@proteinaceous.net.

## Conflicts of Interest

All authors are involved in the commercialization of software for proteoform characterization.

## Data Availability

Data sharing is not applicable to this article as no datasets were generated or analyzed during the current study.
